# Evaluation of four machine learning methods in predicting orthodontic extraction decision from clinical examination data and analysis of feature contribution

**DOI:** 10.3389/fbioe.2024.1483230

**Published:** 2024-10-14

**Authors:** Jialiang Huang, Ian-Tong Chan, Zhixian Wang, Xiaoyi Ding, Ying Jin, Congchong Yang, Yichen Pan

**Affiliations:** ^1^ Department of Orthodontics, Shanghai Stomatological Hospital and School of Stomatology, Fudan University, Shanghai, China; ^2^ Shanghai Key Laboratory of Craniomaxillofacial Development and Diseases, Fudan University, Shanghai, China; ^3^ School of Stomatology, Fudan University, Shanghai, China; ^4^ School of Medical Technology, Shanghai University of Medicine and Health Sciences, Shanghai, China; ^5^ Department of Cariology and Endodontology, College of Stomatology, Shanghai Ninth People’s Hospital, Shanghai Jiao Tong University School of Medicine, Shanghai, China; ^6^ National Clinical Research Center for Oral Diseases, National Center for Stomatology, Shanghai, China; ^7^ Shanghai Key Laboratory of Stomatology and Shanghai Research Institute of Stomatology, Shanghai, China; ^8^ Department of Oral and Maxillofacial-Head Neck Oncology, College of Stomatology, Shanghai Ninth People’s Hospital, Shanghai Jiao Tong University School of Medicine, Shanghai, China

**Keywords:** orthodontic treatment, tooth extraction decision, decision tree, machine learning, cross validation

## Abstract

**Introduction:**

The study aims to predict tooth extraction decision based on four machine learning methods and analyze the feature contribution, so as to shed light on the important basis for experts of tooth extraction planning, providing reference for orthodontic treatment planning.

**Methods:**

This study collected clinical information of 192 patients with malocclusion diagnosis and treatment plans. This study used four machine learning strategies, including decision tree, random forest, support vector machine (SVM) and multilayer perceptron (MLP) to predict orthodontic extraction decisions on clinical examination data acquired during initial consultant containing Angle classification, skeletal classification, maxillary and mandibular crowding, overjet, overbite, upper and lower incisor inclination, vertical growth pattern, lateral facial profile. Among them, 30% of the samples were randomly selected as testing sets. We used five-fold cross-validation to evaluate the generalization performance of the model and avoid over-fitting. The accuracy of the four models was calculated for the training set and cross-validation set. The confusion matrix was plotted for the testing set, and 6 indicators were calculated to evaluate the performance of the model. For the decision tree and random forest models, we observed the feature contribution.

**Results:**

The accuracy of the four models in the training set ranges from 82% to 90%, and in the cross-validation set, the decision tree and random forest had higher accuracy. In the confusion matrix analysis, decision tree tops the four models with highest accuracy, specificity, precision and F1-score and the other three models tended to classify too many samples as extraction cases. In the feature contribution analysis, crowding, lateral facial profile, and lower incisor inclination ranked at the top in the decision tree model.

**Conclusion:**

Among the machine learning models that only use clinical data for tooth extraction prediction, decision tree has the best overall performance. For tooth extraction decisions, specifically, crowding, lateral facial profile, and lower incisor inclination have the greatest contribution.

## 1 Introduction

Whether to extract teeth is one of the most important decisions in orthodontic treatment planning. Its frequency has fluctuated over the years, the extraction percentage was 30% in 1953, reached 76% in 1968, and declined to 28% in 1993, this variation was due to considerations in outcome stability, facial esthetics, and technological changes (Proffit, 1994). To date, scholars have been studying and exploring orthodontic extraction decisions to obtain healthier, more stable and more esthetic orthodontic outcomes (Jackson et al., 2017; Proffit, 1994). Occlusion, stability and esthetics are the three goals for a successful treatment plan, but no single rule can give the orthodontist a simple way to decide how to reach these goals, the extraction decision is multi-factorial, involving crowding (Janson et al., 2014; Boley et al., 2003), overjet and overbite (Janson et al., 2003), Bolton ratio (Hasija et al., 2014), Angle and skeletal classifications (Ker et al., 2008), transverse dimension (midline discrepancy, facial asymmetries) (Chang et al., 2011), incisors angulation, presence of root resorption (Maués et al., 2015), soft-tissue profile (Konstantonis et al., 2013), etc. The extraction plan embodies the experience and wisdom of orthodontists, which is difficult and confusing for young or general practitioners (Liu et al., 2021).

With the popularization of big data and artificial intelligence, more and more studies are trying to use machine learning algorithms to assist in extraction decision-making (Khanagar et al., 2021). Commonly used machine learning methods in decision prediction include linear regression (Köktürk et al., 2024), tree models (Köktürk et al., 2024; Etemad et al., 2021; Suhail et al., 2020), support vector machines (Köktürk et al., 2024), neural networks (Köktürk et al., 2024; Jung and Kim, 2016; Li et al., 2019; Xie et al., 2010), etc. from simple to complex. They usually included model measurements and cephalometric data to train the models. Different validation strategies for over-fitting were applied in the previous studies. We took categorical variables that are often recorded in clinical diagnosis, so we chose tree models, support vector machines, and neural networks. Since the number of variables in this study is not very large, we hope to include features to the maximum extent, so we chose the random forest while also trying the decision tree.

The current study aimed to predict tooth extraction decision based on four machine learning methods and analyze the feature contribution. This study invited senior specialists of the orthodontic department of Shanghai Stomatological Hospital to note down the diagnosis and tooth extraction plan of the patients, and applied four machine learning method for prediction of extraction decision-making. We used cross-validation to measure the generalization ability of the model and avoid over-fitting, and calculated feature contribution to explain the key variables that clinicians value when determining extraction planning.

## 2 Materials and methods

### 2.1 Data collection

The clinical materials were collected from consecutive patients visiting the department of orthodontics of Shanghai Stomatological Hospital from 2018 to 2020, including pre-treatment plaster models, clinical examination data, and treatment plans for malocclusion. Plaster models were scanned and stored in STL format.

Two senior doctors were asked to fill in a standardized form to record the patient’s Angle classification, skeletal classification, maxillary and mandibular crowding, overjet, overbite, upper and lower incisor inclination, vertical growth pattern, lateral facial profile. Angle’s classification, crowding, overbite and overjet were measured from dental models. And lateral facial profile was observed from facial photographs. Skeletal classification, upper/lower incisor inclination, and vertical growth pattern were measured in the cephalometric radiographs, using Steiner analysis (SNA SNB ANB U1-SN U1-NA L1-NB MP-SN), Tweed analysis (FMA IMPA FMIA), Wit’s appraisal, Ricketts’s analysis (lower lip to E plane). Experts’ decisions were comprehensive judgments based on the combination of objective measurement indicators and clinical observations.

As for the extraction decisions, only the consensus of the two experts was recorded, otherwise a third expert was invited and the majority opinion prevailed. An endodontic expert was invited to evaluate the preservation value of the residual crown. We marked the non-extraction cases, and then for the extraction cases, we recorded the specific teeth that are to be extracted, although the prediction of the extraction pattern was not involved in this study. The research plan has been approved by the Institutional Review Board of Shanghai Stomatological Hospital [Hu Kou Fang Lun Shen (2017) No. 0005]. Written informed consent was obtained from all the participants.

### 2.2 Inclusion and exclusion criteria

(1) Complete permanent dentition;
(2) No congenitally missing teeth or impacted teeth;
(3) The tooth extraction in the treatment plan is the classic extraction mode, that is, symmetric extraction of premolars;
(4) Complete pre-treatment model, clinical examination data and orthodontics treatment plan information;
(5) Informed consent signed by the patient or the parent (for teenager under 18).

**FIGURE 1:**
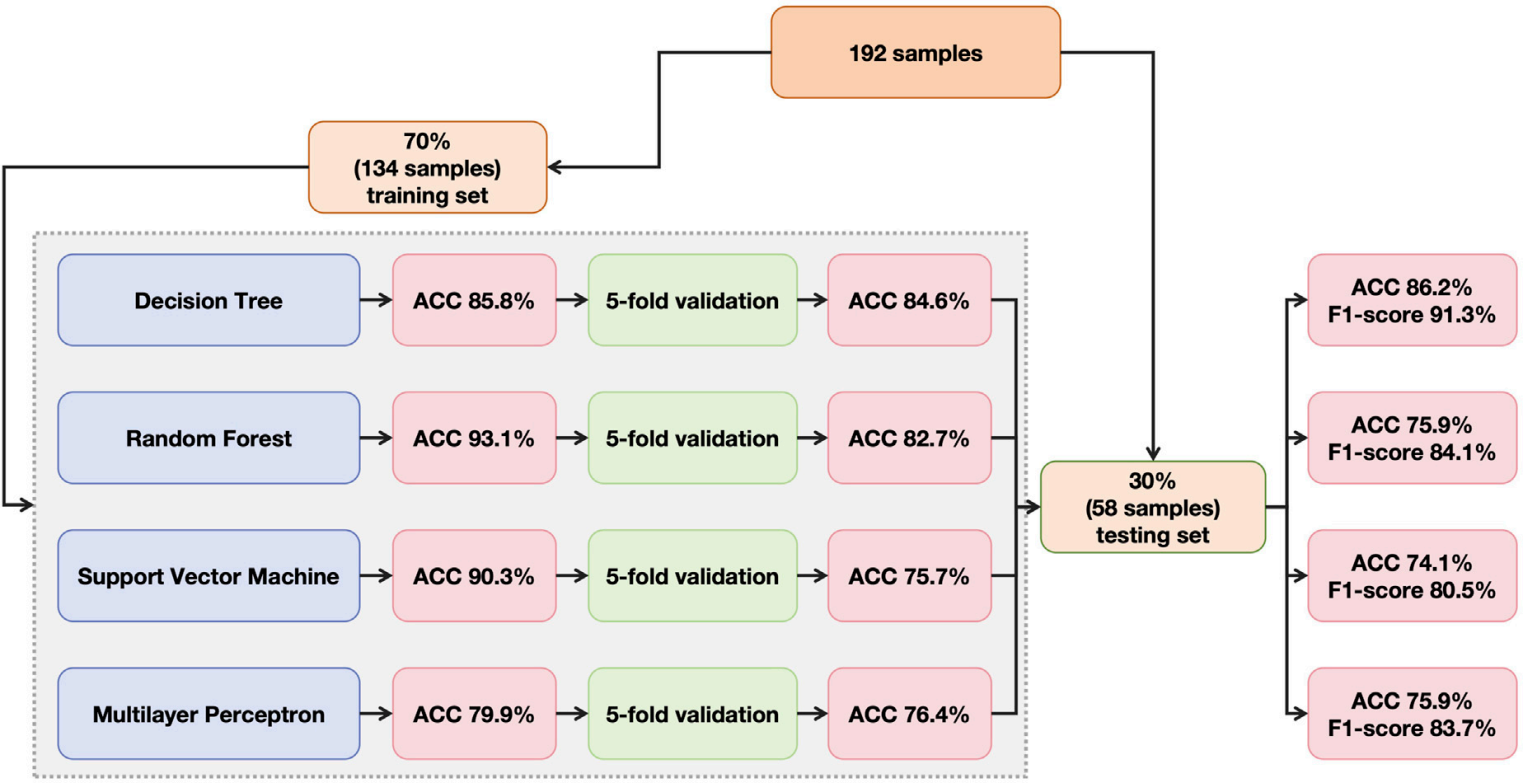
Flowchart of data allocation, model training, cross-validation and model testing.

In total, 192 patients with complete information were included, among whom, 30% were randomly selected as testing set and the rest were divided as training set (Figure 1).

### 2.3 Model training

Four machine learning frameworks were applied for prediction of extraction and non-extraction planning, including decision tree (DT), random forest (RF), support vector machine (SVM) and multilayer perceptron (MLP) (Figure 1).

Decision Trees are built by continuously splitting the data into binary nodes that acquire the largest information gain until the terminal node outputs the predictions of classification (Suhail et al., 2020).

Random Forest is an ensemble of decision trees. Each decision tree performs the same classification prediction individually, and the final classification, or output, is determined by taking the most common predictions for discrete variables or the average of predictions for continuous variables (Etemad et al., 2021). The number of trees was set to 1,000 in our study.

Support Vector Machine (SVM) can efficiently perform a non-linear classification using what is called the kernel trick, representing the data only through a set of pairwise similarity comparisons between the original data points using a kernel function, which transforms them into coordinates in the higher dimensional feature space (Leavitt et al., 2023).

Multilayer Perceptron (MLP) is one of the simplest forms of artificial neural network (ANN), composed of input layer, output layer and hidden layer(s). Each layer consists of multiple nodes, called neuron, fully connected with nodes at adjacent layers (Etemad et al., 2021).

Due to the limited number of features, we removed data containing missing values. And convert continuous data into discrete data using the commonly used classification in orthodontics. Since age and gender nearly had no contribution (0%) to the model in the preliminary experiment, they were removed from the features in the formal study. Finally, nine feature variables were Angle’s classification, skeletal classification, crowding, overbite, overjet, upper and lower incisor inclination, vertical growth pattern and lateral facial profile (Table 1) and one classification variable was extraction/non-extraction decision.

**TABLE 1:** Definitions of nine feature variables.

Variables	Labels	Definitions
Angle’s classification	1	Class I
	2	Class II
	3	Class III
Skeletal classification	1	Class I
	2	Class II
	3	Class III
Crowding	0	Less than 2 mm
(Maximum of upper and lower crowding)	1	2–4 mm
	2	4–8 mm
	3	Over 8 mm
	4	Spacing
Overbite	0	Less than 1/3 overlap
	1	1/3–1/2 overlap
	2	1/2–2/3 overlap
	3	Over 2/3 overlap
	4	Open bite
Overjet	0	Less than 3 mm
	1	3–5 mm
	2	5–8 mm
	3	Over 8 mm
	4	Edge to edge
	5	Cross bite
Upper incisor inclination	1	Buccal inclination
	2	Vertical
	3	Lingual inclination
Lower incisor inclination	1	Buccal inclination
	2	Vertical
	3	Lingual inclination
Vertical growth pattern	1	Hyperdivergent
	2	Normal
	3	Hypodivergent
Lateral facial profile	1	Convex
	2	Straight
	3	Concave

Since there is a difference between the proportion of tooth extraction cases (144 cases) and non-tooth extraction cases (48 cases) in the dataset, in order to deal with the problem of imbalanced classification, we adopted the threshold moving method (Collell et al., 2018). According to the ROC curve of the training set, we select the optimal classification threshold to prevent the model from being “occupied” by the classification with more data.

### 2.4 Cross-validation

Cross-validation is often used to measure the generalization ability of a model (Jonathan et al., 2000; Karkkainen, 2014). In order to ensure adequate number of samples in the cross-validation set, this study used a five-fold cross-validation method. It divided the dataset into five parts, taking turns to use four of them as training set and one as validation set. In each validation, the four training sets will generate a model, and the validation set will be input into the model for classification task. The proportion of the number of times the classifier decision matches the ground truth to the total number of tests is calculated as the accuracy of each validation. The average accuracy of the five validations is taken as the accuracy of a five-fold cross-validation.

### 2.5 Model testing

**FIGURE 2:**
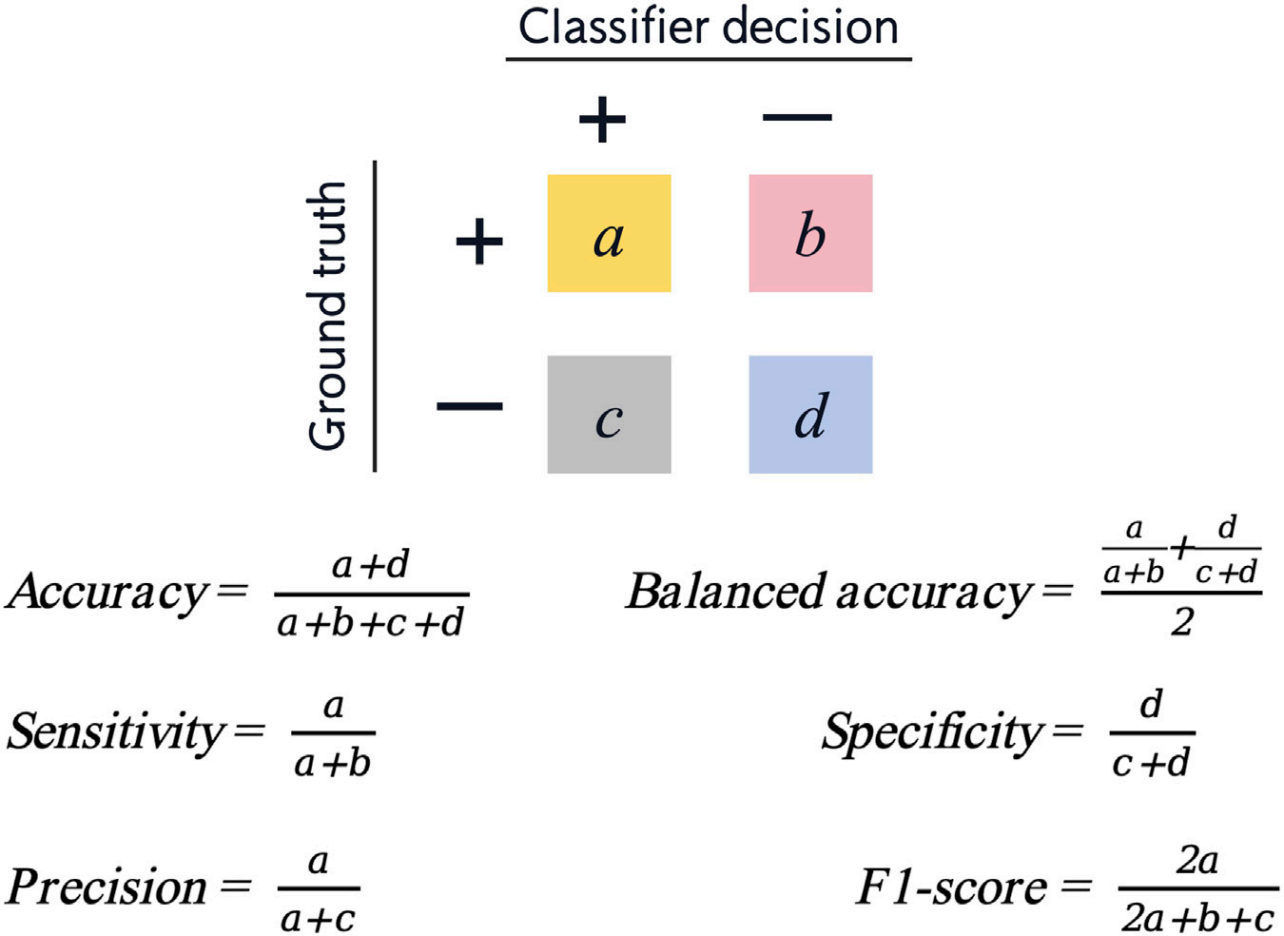
Confusion matrix and six measurements for evaluation of model performance (positive cases represent extraction classification and negative cases represent non-extraction classification).

In the dataset, 30% (58 samples) of all patients were randomly selected as the testing set. The above four machine learning models were used for testing. The results of the testing set were represented by confusion matrix, and the following indicators were calculated (Figure 2):

• Accuracy: correctly classified data points in the testing set.
• Balanced accuracy: the average of sensitivity and specificity.
• Sensitivity/recall: positive data points correctly classified as positive.
• Specificity: negative data points correctly classified as negative.
• Precision: data points classified as positive that are actually positive.
• F1-score: the harmonic mean of precision and sensitivity. Precision and sensitivity may affect each other. Although it is ideal for both to be high, but in reality, it is often the case that the precision is high and the sensitivity is low, or otherwise. Therefore, F1-score is an indication of both at the same time.

We also plotted the receiver operating characteristic (ROC) curve to visually demonstrate the performance of the model and calculated the area under the curve (AUC).

### 2.6 Feature contribution

Feature contribution in tree models can be measured by the number of times a feature participates in building the tree and the cumulative value of information gain when it is used as a split node. In the process of building a tree, the more times the feature is used to split nodes, the more important role the feature plays in the decision process. Cumulative value of information gain represents the amount of reduction in information entropy when the feature is used for node splitting. The greater the information gain, the greater the contribution of the feature to improving the predictive ability of the model. In addition, early participation of a feature (at the upper nodes of the tree) is usually considered more important than late participation and the cumulative sum was calculated in a weighted manner to reflect the contribution of the features.

## 3 Results

### 3.1 Descriptive statistics

**FIGURE 3:**
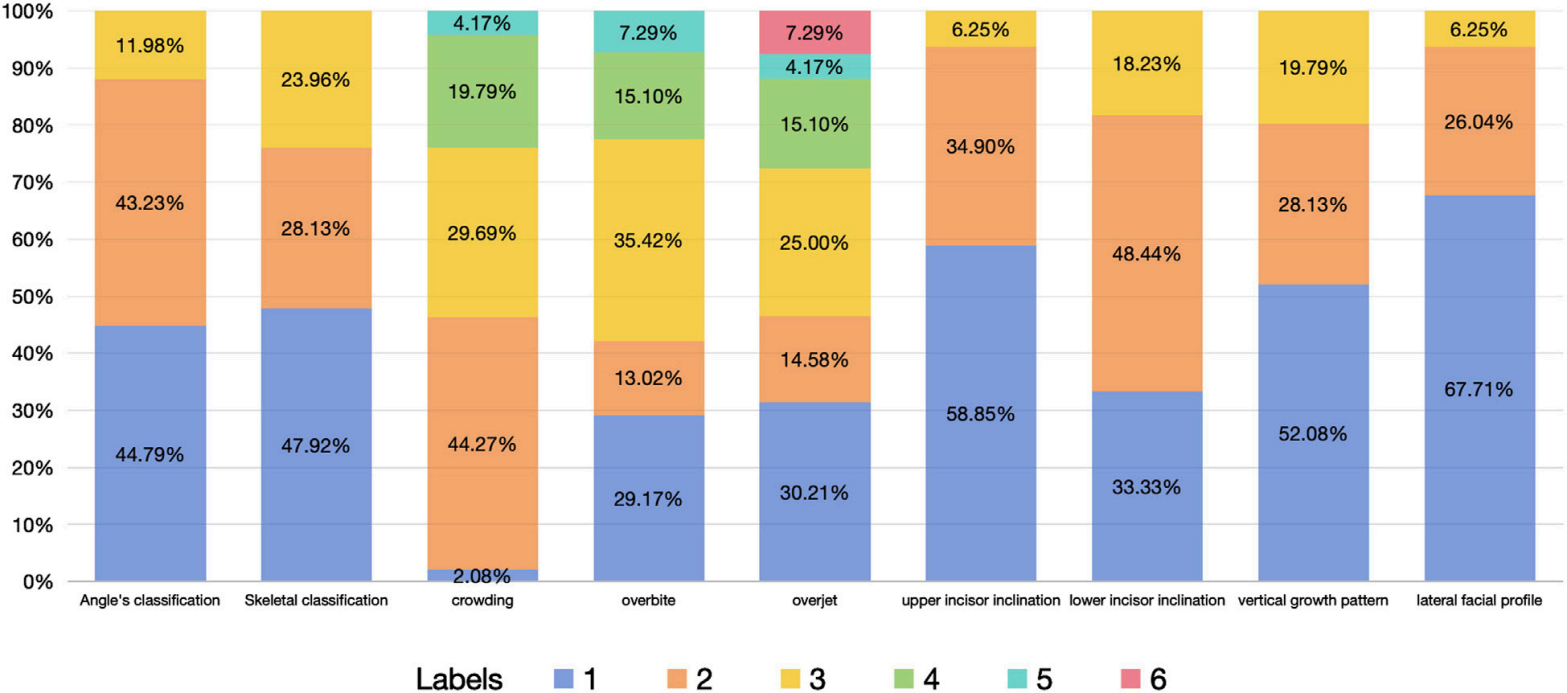
Proportion of each feature variables.

The dataset of this study includes 48 non-extraction cases and 144 extraction cases. The proportion of each feature variables is shown in Figure 3.

Since crowding often changes in similar directions in the upper and lower dentitions, we combined it into one variable-crowding, by calculating the maximum of crowding in upper and lower dentitions. However, the inclination of the upper and lower anterior teeth can appear completely opposite trends, such as buccal inclination in upper incisor and lingual inclination in lower incisor, so they were considered separately in this study. Open bite and deep overbite reflect different degrees of vertical discrepancy of the upper and lower anterior teeth in different directions, so they were combined into one variable as overbite (Table 1; Figure 3).

### 3.2 Evaluation of model accuracy

**FIGURE 4:**
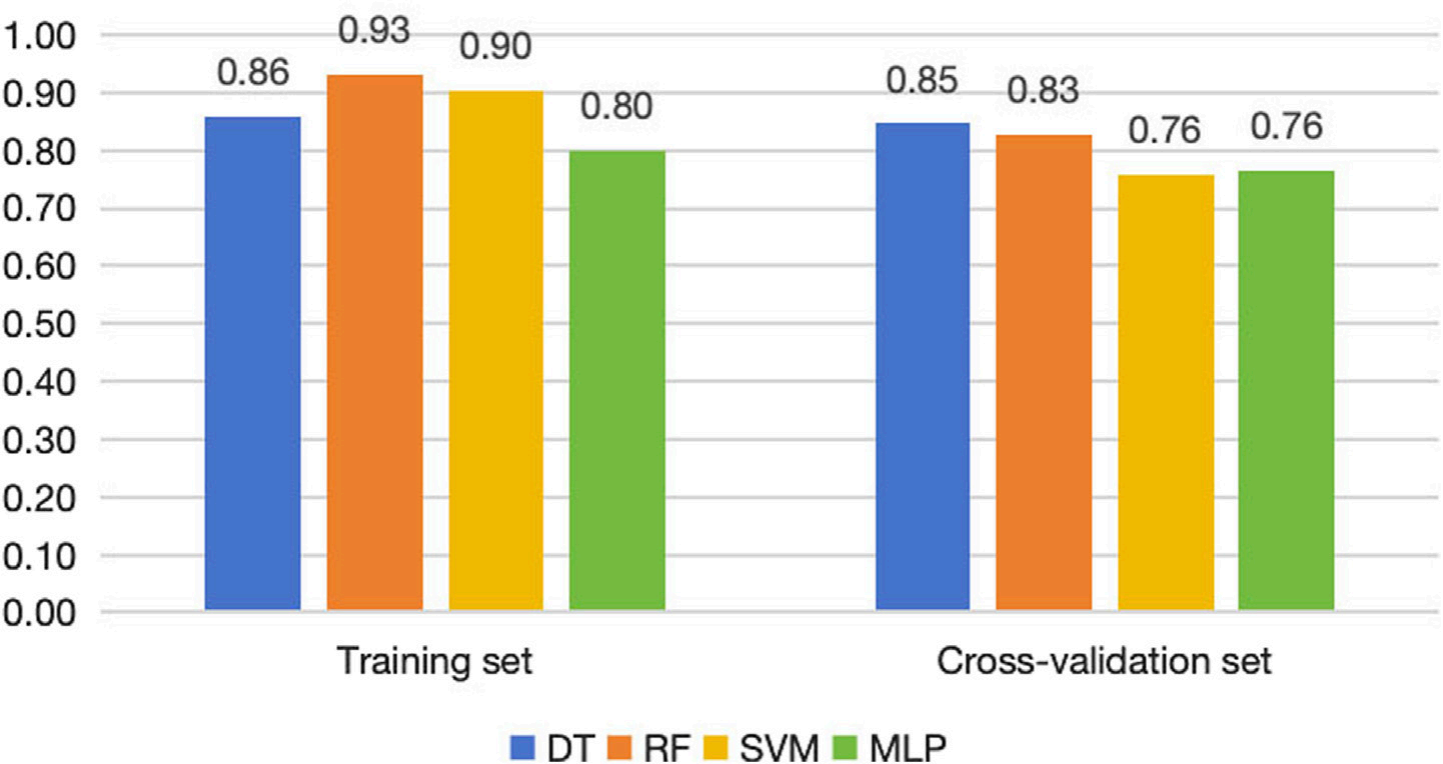
Accuracy of the four machine learning models in training sets, cross-validation sets and testing sets.

The accuracy of the four models in the training set and cross-validation set is shown in Figure 4. The accuracy of the four models in the training set ranges from 80% to 93%, and in the cross-validation set, the decision tree and random forest had higher accuracy. We can evaluate the generalization ability of the model by comparing the difference between the accuracy of cross-validation set and training set. Generally, over-fitting is often seen if the accuracy in the cross-validation set is significantly lower than that in the training set, but there is not a definite border (Xu and Goodacre, 2018). In this study, random forest and SVM showed a tendency of, but not absolute, over-fitting. The performance of decision tree and MLP was acceptable, and the accuracy of decision tree was greater than that of MLP.

**FIGURE 5:**
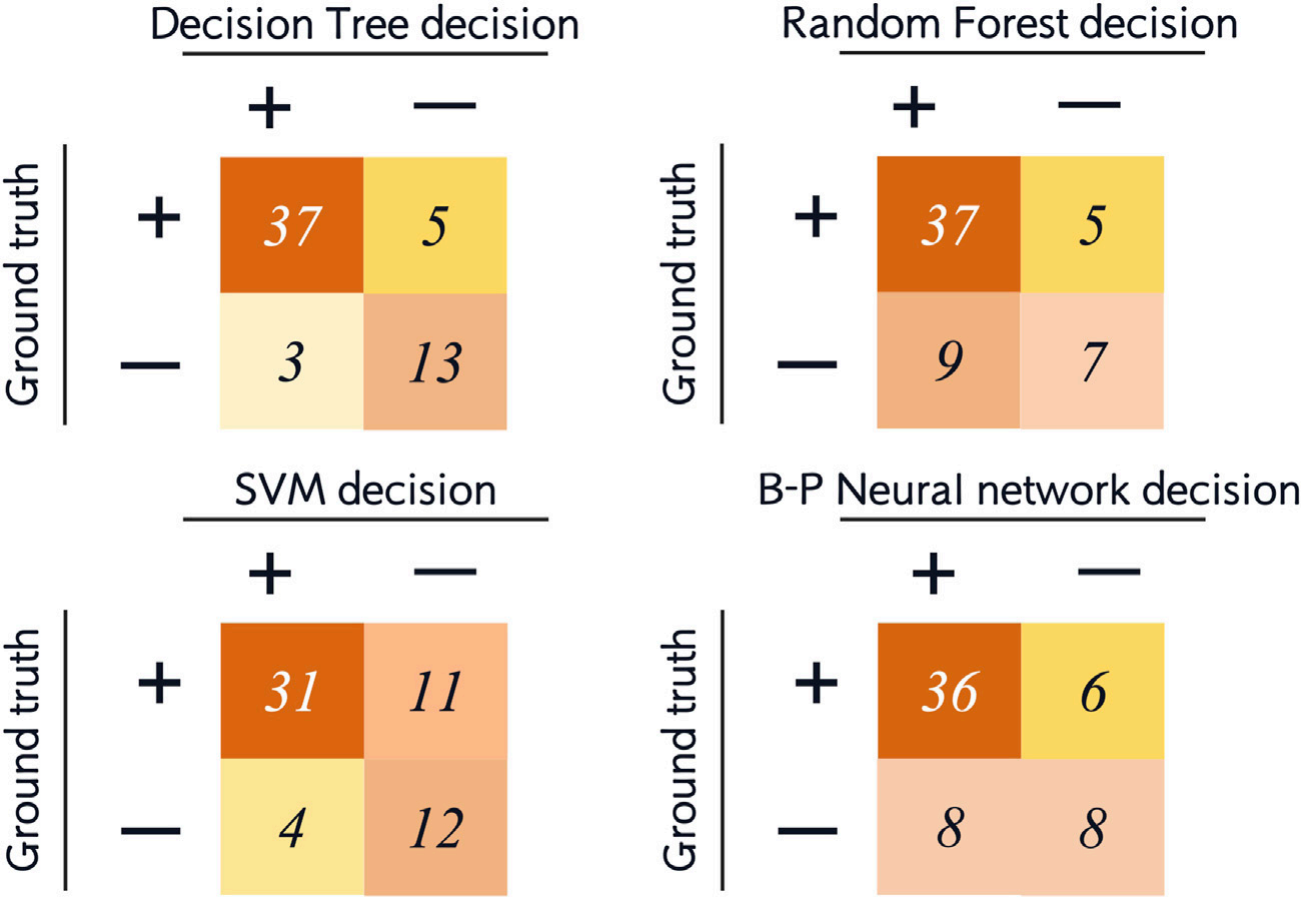
Confusion matrix of the testing sets of the four machine learning models.

**FIGURE 6:**
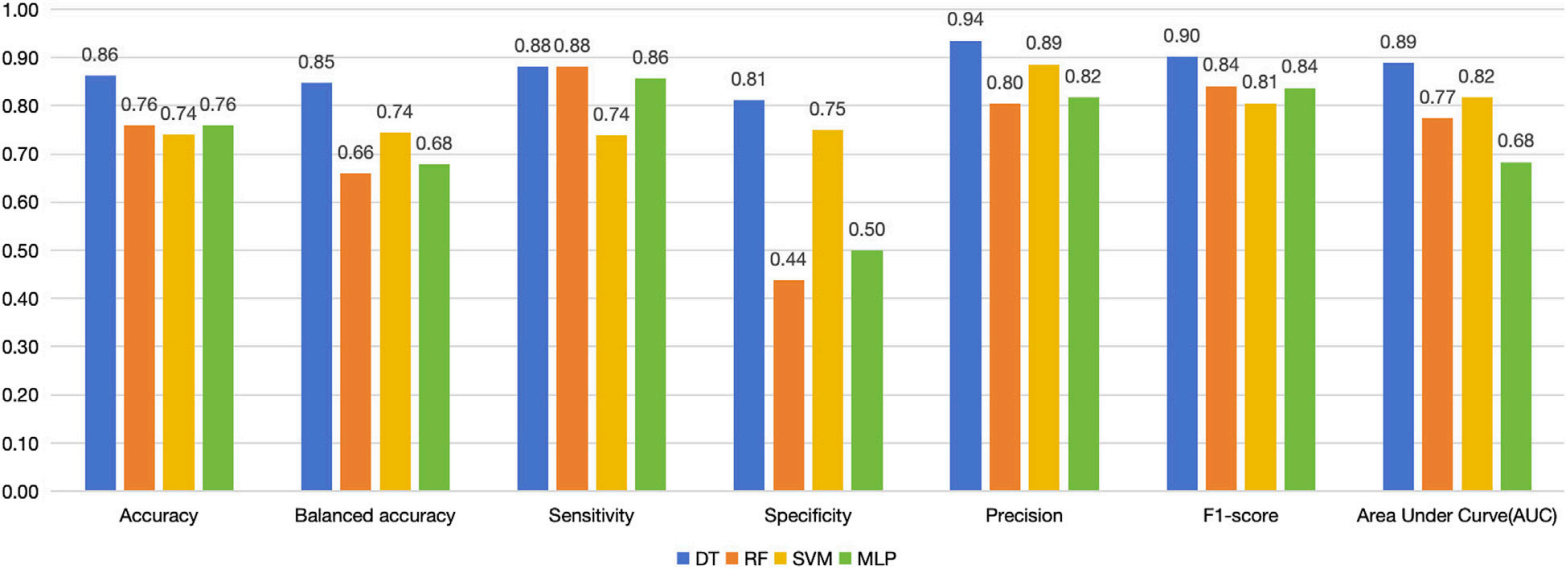
Performance of the four models in testing set.

For the testing set, we list the confusion matrices of the four models, in which, positive cases represent extraction classification and negative cases represent non-extraction classification (Figure 5). In the random forest and SVM, the accuracy of the testing set was also significantly lower than the training set (Figures 4, 6). Among the performance of these four models, the decision tree showed the best overall prediction performance with the best accuracy, balanced accuracy, specificity, precision and F1-score (Figure 6). It is worth noting that in a more detailed analysis, RF and MLP showed a low specificity, indicating that they tended to classify too many non-extraction cases into extraction group (Figure 6).

**FIGURE 7:**
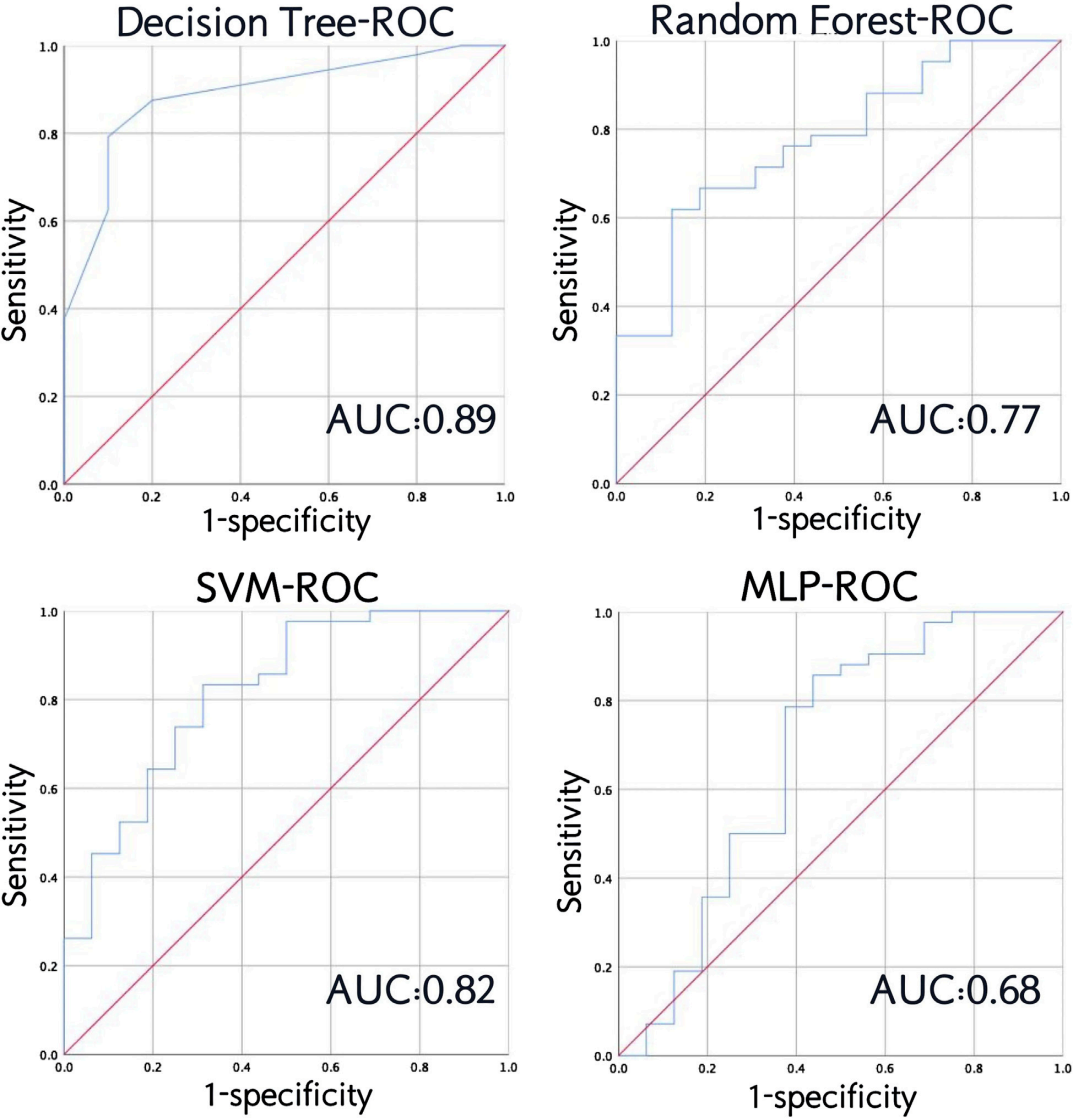
Receiver operating characteristic (ROC) curve and area under the curve (AUC) of the four models in testing set.

From the ROC curve, we can see that the decision tree model performs best, followed by SVM, and MLP is the weakest (Figure 7).

### 3.3 Evaluation of feature contribution

**FIGURE 8:**
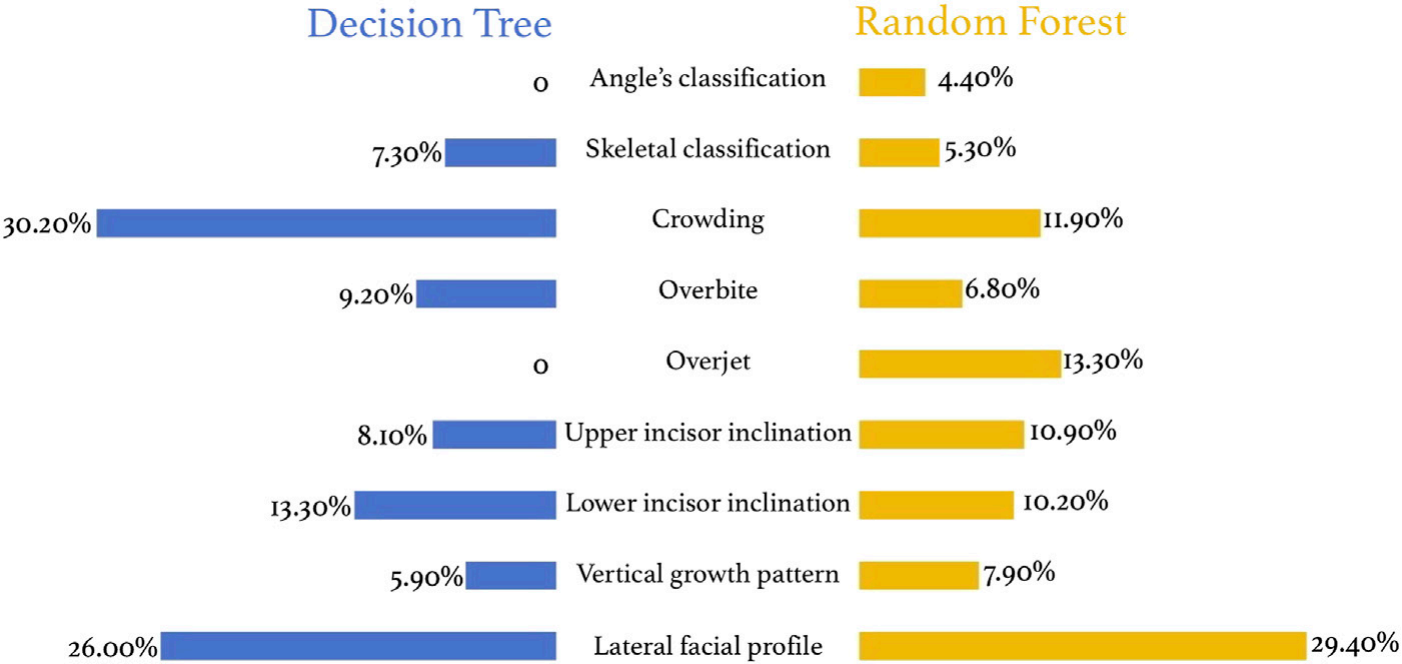
Feature contribution in decision tree and random forest models.

The tree model has strong interpretability and is significantly better than support vector machines and neural networks. In the decision tree model, crowding is the most important indicator for tooth extraction (30.20%), followed by lateral facial profile (26.00%), lower incisor inclination (13.30%), overbite (9.20%), upper anterior lip inclination (8.10%), and skeletal classification (7.30%) (Figure 8). In the random forest model, the most important indicator is lateral facial profile (29.4%), followed by overbite (6.80%), crowding (11.90%), upper (10.9%) and lower incisor inclination (10.20%), vertical growth pattern (7.90%), etc. (Figure 8).

**FIGURE 9:**
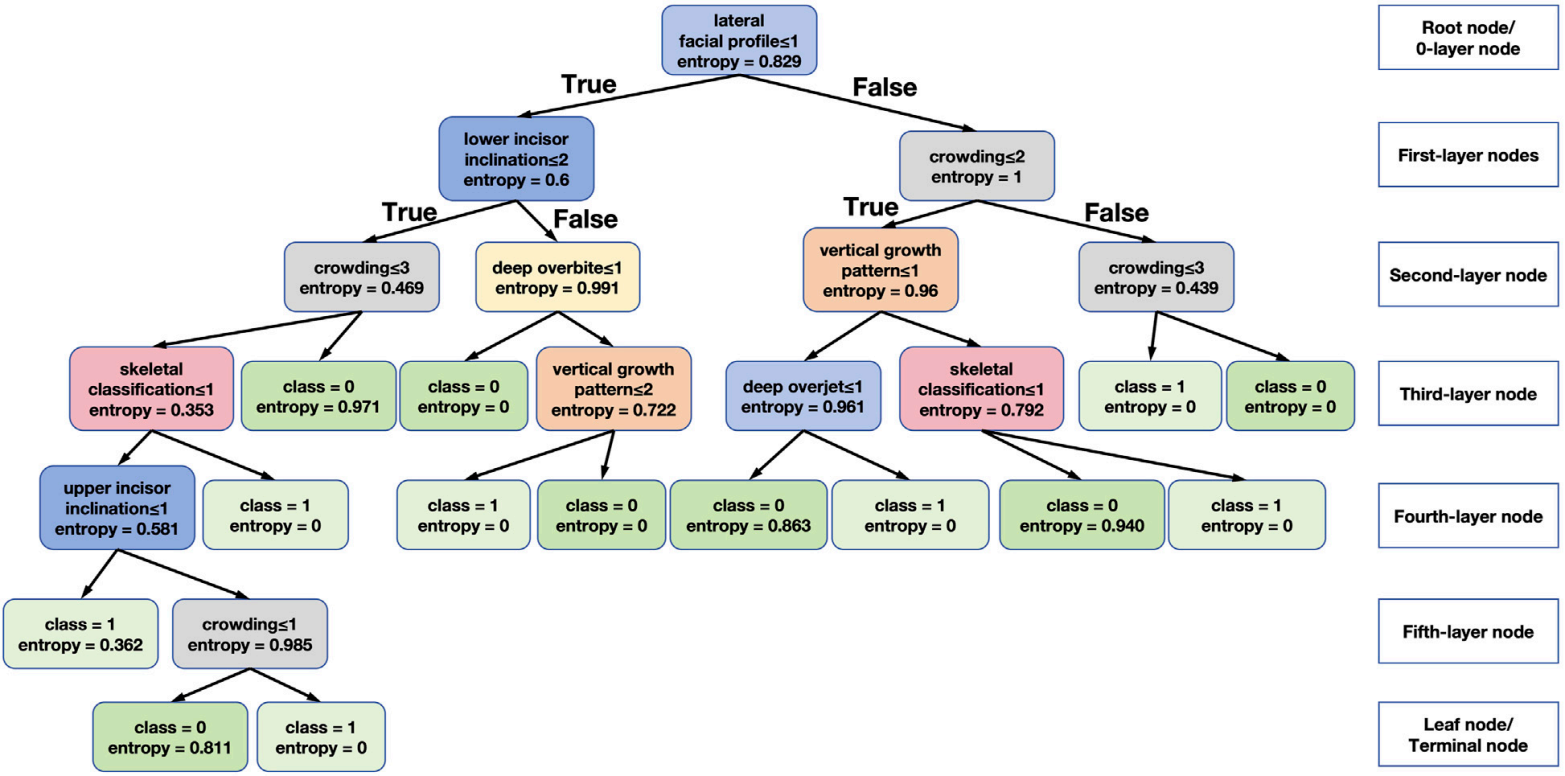
One example of a decision tree generated in our study.

Figure 9 shows one of the decision tree models generated in this study, which facilitates intuitive analysis of the specific role of each feature. The feature selection standard of the decision tree in this study is set to information gain (entropy). Entropy represents the uncertainty of the information of the node, taking a value between 0 and 1. The larger the entropy, the greater the uncertainty. That is to say, for the training set of this tree, the closer the entropy is to 0, the greater the confidence of this classification.

The root node, or 0-layer node, used in the tree in Figure 9 are lateral facial profile, upper and lower incisor inclination, crowding, overbite, vertical growth pattern, skeletal classification. The root node is the lateral facial profile. The model is divided into two categories according to straight, convex and concave lateral facial profile. However, it is unable to make a tooth extraction prediction based on the lateral facial profile alone, so node splits into two branches as first-layer nodes. A more detailed analysis shows that the most commonly used feature was crowding (grey box in Figure 9), and when we look at the most left and right boxes in the second-level nodes, if the crowding score was between 1 and 3 (larger than 2 mm crowding), it tends to extraction decision, and when it is greater than 3 or equal to 4 (spacing), it tends to non-extraction decision. For vertical growth pattern (orange box in the third-level node), a score over 2 (hypodivergent) indicates non-extraction decision. Moreover, Class II and Class III classification (pink box, scored 2 and 3) points to tooth extraction and buccal incisor inclination (blue box in the fourth-level node, scored 1) suggests the tendency to tooth extraction.

## 4 Discussion

Orthodontic treatment planning embodies experience and wisdom of orthodontists, and the formulation of tooth extraction plans is one of the most critical decisions in orthodontic practice, and it is also one of the biggest challenges for young doctors and general practitioners (Liu et al., 2021). Although there are many factors that may influence orthodontic tooth extraction decisions. For most orthodontic cases, orthodontic experts have relatively consistent judgments on tooth extraction planning (Suhail et al., 2020; Xu and Huang, 2002). In this context, it is meaningful to perform automatic decision-making assisted by machine learning.

Because natural prevalence of different categories of malocclusion differs among different populations (Lombardo et al., 2020). At the same time, patients’ motivation to seek orthodontic treatment also varies. For example, Chinese patients may have stronger will to correct convex facial profile (Yin et al., 2014). Therefore, each category of malocclusion in this study cannot be completely balanced. If the proportion of each category of malocclusion is calibrated during the inclusion process, randomization cannot be ensured, and it may be difficult for the model to adapt to clinical reality.

Classification algorithm is an important technology in data mining. In this study, the decision tree was very effective in classifying the sample set. In orthodontic tooth extraction decisions, random forests usually perform better than decision trees (Prasad et al., 2022; Etemad et al., 2021). Random forests may randomly select a certain proportion of features to be included in each model (Belgiu and Dragut, 2016). When the scale of the decision tree is too large, it may lead to over-fitting. Therefore, this study appropriately limited the depth and number of nodes of the tree during the parameter adjustment process. Random forest is suitable for most types of datasets, but in this study, no matter how the parameters are adjusted, random forest still shows some over-fitting tendency. Neural networks are more suitable for large and complex datasets. In the analysis of the confusion matrix, RF, SVM and MLP all showed low specificity (<0.4) at the first experiment, showing a strong preference to the tooth extraction decision. This may be attributed to the imbalance of the classification variable of the dataset. Among the consecutive patients in our hospital, the number of extraction treatment is larger than non-extraction, resulting in more positive cases in the testing set. Therefore, we adopted threshold moving method to select an optimal classification threshold (Collell et al., 2018). As a result, the specificity improved in SVM. Still, in RF and MLP models, adding the judgment of the extraction decision may improve the accuracy, resulting in decision bias. However, the decision tree shows better discernment of non-extraction cases. The more complex the model, the greater the possibility of over-fitting. Decision tree is more resistible to uneven category in this study. Decision tree is based on information entropy for classification, so it essentially looks at the correlation between features and categories. Even if there is little data in this category, as long as its correlation with the features is strong, it will not be misclassified. Random forest has a step of random sampling of data. If the category distribution is uneven, this class may not exist in some sub-sampling, which will affect the results.

There is occasionality in the generation of the model, and high accuracy in a single test does not mean stable performance of the model. In order to evaluate the generalization of the model, we conducted cross-validation. In cross-validation, each sample will appear once in the validation set, therefore, the model needs to have good generalization ability for all samples in the dataset in order to achieve a high average cross-validation accuracy (Jonathan et al., 2000; Karkkainen, 2014). Xie et al. (2010) used an artificial neural network model to predict the extraction plan and obtained an accuracy of 100% in the training set and 80% in the testing set, indicating over-fitting of the model. Köktürk et al. (2024) used multiple machine learning methods for tooth extraction decision-making, and the best algorithm was Gradient Boosted Trees, with an accuracy of 83.3%. We obtained an accuracy of 81.5% using MLP. Li et al. (2019) and Jung and Kim (2016) both used deep neural networks to train tooth extraction prediction models on samples of 156 and 302 patients, respectively, achieving an accuracy rate of over 93%, but their study did not specify the validation method they used. However, Köktürk et al. (2024), Etemad et al. (2021) and the current study also used the MLP method, but none of the accuracy exceeded 82%. They further divided the dataset into a correctly predicted group and an incorrectly predicted group according to the classification results of this preliminary model, and used the same method to calculate the accuracy, which was improved in both groups (Etemad et al., 2021). However, such a division has no clinical implication, and therefore impossible to find the corresponding external testing set, so it cannot be said that this result can be generalized. Interestingly, Etemad et al. (2021) found that the accuracy of the model obtained by using 117 variables did not improve compared with that using 22 variables. Our study only used 9 variables and obtained an accuracy exceeding their study. Suhail et al. (2020), unlike previous studies, significantly reduced the number of diagnostic features (nineteen) and demonstrated that finite feature sets and machine learning algorithms can accurately predict the extraction process. The ensemble of simpler models outperforms more complex models, like neural network.

There is a huge difference in the tooth extraction rate between Eastern and Western people. The tooth extraction and correction rate of Western people is around 10%–30%, much lower than that of East Asian people (Jackson et al., 2017). Economic, psychological, physiological and anatomical conditions may cause various extraction rate and complex influencing factors among different countries (Del Real et al., 2022), resulting in distinct difficulty of model training. Del Real et al. (2022) obtained an accuracy of more than 90% in one of their models in a Chilean population, but in their sample, cases of skeletal class I accounted for 65%, and skeletal class III only 6%, and the average angle cases were also close to 50%, the proportion of normal facial type was significantly higher than in our study.

Tooth extraction plan can be affected by the patient’s own factors. Some patients strongly resist tooth extraction, which may lead to the actual number of tooth extraction classifications in the final included data being less than the number of tooth extraction classifications that should be, which may be one of the reasons for the misclassification of the prediction model. Therefore, in this study, we recorded the experts’ preferred plans rather than the actual plans in the final dataset to predict the tooth extraction plan.

The interpretability of the tree models significantly outperforms other models like deep neural networks. Since the decision tree model worked better in this study, and a tendency of over-fitting occurred in the random forest, the results of the decision tree should be referred to in the feature contribution analysis. The most important indicators of extraction treatment were crowding, lateral facial profile, and lower incisor inclination. These features can all reflect the tooth-bone volume discrepancy. On the other hand, indicators such as Angle’s classification and overjet may reflect the sagittal discrepancy of maxilla and mandible and does not influence orthodontic extraction decisions from our study. Li et al. (2019) found out that upper and lower crowding, and U1-NA°, are the three most important features. Su et al. (2022) studied the tooth extraction pattern of patients with skeletal Class II average angle and deep overjet and showed that deep overjet and distal molar relationship are the main reasons for tooth extraction in Class II patients. Guo et al. (2014) founded that lower anterior crowding, molar relationship, and growth pattern were the three most relevant influential factors to the extraction decisions for Angle’s class II division 1 malocclusions. The above three studies all found that crowding is the most important influencing factor in determining tooth extraction decisions, but other factors are slightly different from this study. However, Bishara et al. (1995) pointed out that tooth size-arch length discrepancy and lip protrusion are the main reference factors for tooth extraction correction, which is highly consistent with the results of our study.

It should be noted that the purpose of introducing machine learning to assist in the classification of orthodontic tooth extraction plans is not to pursue a wiser judgment than that of orthodontists in critical cases, because whether it is expert judgment or machine learning classifier, the plan formulation of critical cases needs to weigh non-orthodontic indicators such as periodontal conditions, patient’s own will, and public aesthetics, rather than just the accuracy as a measurement standard. Machine learning methods should be able to better summarize the logic and ideas of experts, explain the causes and nature of malocclusion, and help doctors formulate more comprehensive treatment plans. Any mathematical principle that affects decision-making cannot be isolated from clinical examination and communication.

## 5 Conclusion

The decision tree algorithm in machine learning outperformed other machine learning models in predicting orthodontic extraction plans, with an average accuracy of 86%. Crowding is the most important factor for experts to decide on extraction treatment, followed by lateral facial profile and lower incisor inclination, indicating that tooth extraction is an important treatment method for tooth-bone volume discrepancy. Clinically, lack of space, protrusive anterior lips and convex facial profile indicates the need for tooth extraction. Machine learning should not replace but help doctors formulate more comprehensive treatment plans.

## Data Availability

The raw data supporting the conclusions of this article will be made available by the authors, without undue reservation.
